# Lipid in microglial biology — from material to mediator

**DOI:** 10.1186/s41232-023-00289-z

**Published:** 2023-07-17

**Authors:** Shota Yamamoto, Takahiro Masuda

**Affiliations:** Division of Molecular Neuroimmunology, Medical Institute of Bioregulation, Kyushu University, Fukuoka, 812-8582 Japan

**Keywords:** CNS, Lipid, Macrophage, Microglia

## Abstract

Microglia are resident macrophages in the central nervous system (CNS) that play various roles during brain development and in the pathogenesis of CNS diseases. Recently, reprogramming of cellular energetic metabolism in microglia has drawn attention as a crucial mechanism for diversification of microglial functionality. Lipids are highly diverse materials and crucial components of cell membranes in every cell. Accumulating evidence has shown that lipid and its metabolism are tightly involved in microglial biology. In this review, we summarize the current knowledge about microglial lipid metabolism in health and disease.

## Background

What are lipids? The most classical definition is “biomolecules that are insoluble in water, but soluble in organic solvents.” However, cumulative knowledge has pointed out the difficulty of correctively defining lipids in that way, due to the presence of soluble lipids bound or attached to hydrophilic chemical structures or proteins at the very least. In addition, this hydrophobic property must be responsible for the uniqueness of lipids in our organisms, in which water-mediated biological activities are dominant. Amphipathic properties of some lipid classes, such as glycerophospholipid (GPL), sphingomyelin (SM), and cholesterol, are suitable to form compact and flexible biological lipid bilayer membranes, which contribute to making intracellular (extracellular) spaces for the birth of cells. Lipids are not directly encoded by genes, but are biosynthesized and metabolized by lipid-related enzymes, or can be supplied through dietary intake. Major lipid categories contain fatty acids, glycerolipids, GPLs, sphingolipids, and sterols. These are categorized based on their structural diversity, such as backbone types, the number and position of carbons, oxygens, double bonds, and others. It is estimated that there are over 10^5^ lipid species in mammals, and the lipid compositions are quite different among tissues [3, 49, 53]. In addition, lipids play pivotal roles not only in membrane formation but also in energy and heat storage, intercellular signaling, and, furthermore, creating microenvironments required for optimal cellular communication by regulating membrane mechanical/chemical properties [3, 21]. Therefore, mutations in or knockout of lipid-related genes often induce severe phenotypes in mice including embryonic or neonatal lethality [27, 56]. Similarly, dysregulation of lipid metabolism or mutations in lipid-related receptors are implicated in various diseases in human, and excellent reviews are available, including well-summarized tables describing the names of the lipid, associated genes, and their related diseases [21, 60]. Hence, lipids are crucial biomolecules in the diverse aspects of biological events.

## Diverse lipids in the central nervous system

Transcriptome and proteome analyses of cells in the central nervous system (CNS), such as brain and spinal cord, have provided beneficial resources cataloging mRNA and protein expressions at diverse conditions in physiological development and disease [34]. In addition, although it has lagged behind such analyses, a recent technological advance of analyzing lipids has enabled to focus on the CNS lipidome. The lipid composition of the CNS substantially differs from the periphery, and even within the brain, region-specific distinct lipid profiles are observed in adult mice [3, 15, 21, 57]. A clear example of the brain regional specificity is the comparison between white and gray matter in the CNS. The white matter contains high levels of cholesterols and plasmalogens which have ether-linked phosphatidylethanolamine, whereas higher levels of phosphatidylserine (PS) and phosphatidylinositol constitute the gray matter [15, 57]. Furthermore, at a cellular level, distinct lipid compositions are evident when comparing neurons, oligodendrocytes, astrocytes, and microglia in vitro, which may also account for the reginal difference. For instance, oligodendrocytes, which form myelin sheaths, are rich in plasmalogens, cholesterols, and sulfatide, whereas neurons comprise high levels of ceramides as well as cholesterols. On the other side, astrocytes are enriched with PS and diacylglycerol, and microglia display high levels of phosphatidylglycerol and SM [15].

The brain is one of the lipid-rich tissues among whole body, as the lipid content accounts for about 50% of its dry weight [54]. However, the brain is strictly separated from peripheral blood by the blood–brain barrier (BBB), raising the question of where brain lipids are provided from. Brain cholesterols are known to be almost independent from the periphery and entirely synthesized within the brain [42, 60]. De novo synthesis of cholesterols in the brain is mainly achieved by astrocytes, with minor contributions by neurons and oligodendrocytes [38]. The astrocytes-derived cholesterols are transferred intercellularly via the forms of lipoprotein particles containing apolipoproteins, such as apolipoprotein E (ApoE), under homeostatic conditions. In addition, the cholesterol synthesis is highly active during development, which is in contrast limited in the healthy adult brain [11, 29]. In regard to glycerolipids, a positive uptake mechanism from peripheral blood involves despite the presence of the BBB. Major facilitator superfamily domain containing 2a (Mfsd2a) was identified as a lysophosphatidylcholine (LPC) transporter expressed in the CNS endothelial cells [37]. These LPC may be recycled as materials for glycerol backbones, fatty acids, and choline, although the fate of LPC after uptake by Mfsd2a remains unknown. On the other hand, mammals lack the ability of de novo synthesizing polyunsaturated fatty acid (PUFA), such as docosahexaenoic acid and arachidonic acid; thus, uptaking PUFA from diet is necessary alternatively. By contrast, saturated and monounsaturated fatty acids such as palmitic acid or oleic acid can be produced in the de novo pathway via fatty acid synthase and stearoyl-CoA desaturases. However, very little is known about the maintenance mechanisms of the brain whole lipidome, which needs a more in-depth approach, such as comprehensive lipidomic analysis, in the future.

## Lipid-related genes in microglia

Microglia are the resident macrophages of the CNS, which occupy the entire parenchyma of the CNS, where various cell types that possess a chemically diverse set of lipids component constitute highly complex 3-dimentional structures [32, 44]. Microglia express several lipid-related genes, and their genetic mutations cause abnormalities in lipid metabolism and lead to inherited diseases. Among them is triggering receptor expressed on myeloid cells 2 (TREM2) that is exclusively expressed in microglia in the CNS. TREM2 is known as an extracellular lipid sensor [5] and mediates myelin debris clearance [43], and the amino acid polymorphisms of *Trem2* cause neurological disorders, such as Alzheimer’s disease and Nasu-Hakola disease [18, 19, 24, 41], although the precise mechanisms by which the mutation causes a neurodegenerative process are not fully understood. ApoE is also expressed in microglia and is drastically upregulated during neurological diseases. Inheritance of ApoE ε4 allele is known as a risk factor for Alzheimer’s disease [8, 46]. Actually, lipid accumulation is observed in the postmortem brain of Alzheimer’s disease patients [10], and microglial dysfunction resulted from *Trem2* mutations or inheritance of ApoE ε4 allele should be involved in these neuropathological phenotypes. Furthermore, hexosaminidase subunit beta (HEXB) is a stably expressed microglia core gene, which encodes the beta subunit of the lysosomal enzyme beta-hexosaminidase that hydrolyzes GM2 gangliosides, synthesized from ceramides. It has been reported that defective enzymatic activity of beta-hexosaminidase induces the accumulation of GM2 gangliosides, resulting in Sandhoff disease, which represents severe neurological dysfunctions [47].

**Fig. 1:**
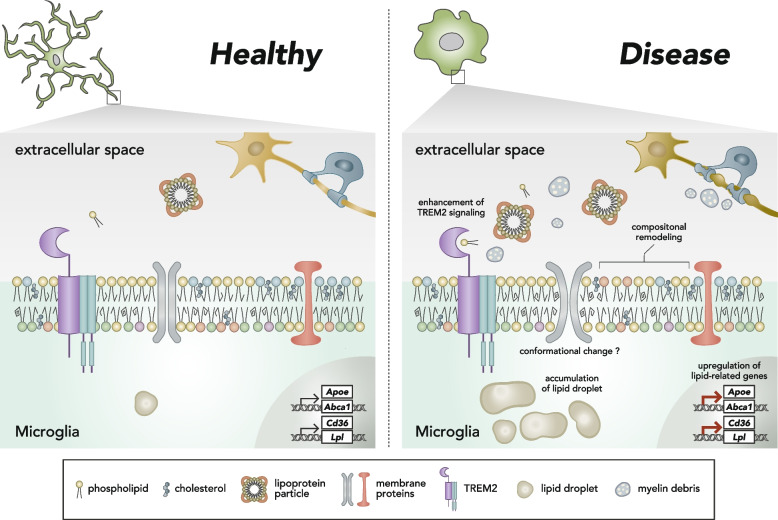
Lipidomic alterations of microglia between healthy and disease conditions. During disease and inflammation, microglial lipidome is drastically changed, which includes upregulation of lipid-related gene expressions (*Abca1*, *Apoe*, *Cd36*, *Lpl*, and others), accumulation of lipid droplet, compositional remodeling of membrane phospholipids, and followed by conformational changes of membrane proteins

Microglia/macrophages drastically modulate the expression of lipid-related genes in a context-dependent manner. During the pathogenesis, microglia concomitantly upregulate expression levels of lipid-related genes (*Abca1*, *Apoe*, *Apoc1*, *Cd36*, and *Lpl*) and undergo proliferation [22, 23, 33, 48], which might indicate that microglia positively shift their lipid metabolic states to utilize lipids for energy source and materials of cell membranes (Fig. 1). Moreover, the TREM2-ApoE pathway drives microglia to be a neurodegenerative state, which exerts enhanced cellular activity [26]. It is also reported that the expression level and activity of sterol regulatory element-binding protein 1 (SREBP1), the key transcription factor required for lipid metabolism, are increased after lipopolysaccharide treatment in macrophages in the periphery. SREBP1-mediated PUFA production is necessary for a phenotypic switch toward a resolving state [40]. Actually, increased levels of fatty acids or compositional changes of GPLs were observed in microglial cell line after lipopolysaccharide or in microglia from disease model mice [4, 7, 39]. Furthermore, in macrophages, compositional remodeling of membrane sterols is occurred in response to bacterial toxins as a host defense mechanism [61]. To sum, microglia not only express genes required for lipid homeostasis but also flexibly control their lipid metabolic status for regulating multiple cellular functions. In subsequent parts of this review, we focus on recent intriguing findings regarding microglial lipid metabolism.

## Lipid accumulation in microglia during aging and diseases

Foamy phagocytes in which abnormal lipid bodies accumulate firstly drew attention in 1970s as a contributor to atherosclerotic lesions [16]. Since then, intracellular lipid accumulation is increasingly recognized as a dynamic player in immune dysfunction in phagocytes [9]. Lipid droplets (LDs), which are lipid storage organelles containing glycerolipid and cholesteryl ester (CE), are observed in microglia of aged human/mouse brains and in mice with demyelination, and these microglia are named “lipid-droplet-accumulating microglia (LDAM)” [6, 30, 39, 50]. Recent multi-omics studies combining lipidomic and transcriptomic approaches have demonstrated dysregulations of microglial lipid homeostasis and their underlying mechanisms during aging and diseases.

### Glycerolipid

Glycerolipids, such as GPL and triacylglycerol/diacylglycerol, consist of a glycerol backbone with fatty acids. In aged human and mouse brains, microglia labeled with BODIPY (a marker of LDs) and perilipin 2 (PLIN2) or PLIN3 (the LD surface proteins) are clearly observed [30, 50]. Interestingly, lipidome profiles of microglial LD from aged mice substantially differ from those of liver LD in levels of each lipid class (glycerolipid, CE, SM, ceramide, and free fatty acid) and chain-length distribution of fatty acids in glycerolipids [30]. These LD-rich aged microglia highly produce reactive oxygen species and proinflammatory cytokines and have lower ability of phagocytosis [30]. In multiple sclerosis (MS) patients, oxidized phosphatidylcholines (OxPCs) (or their reactive antibodies) are found in the brain and cerebrospinal fluid [20, 25, 45]. Although accumulation of OxPCs has been recognized as a marker of oxidative stress, a recent study demonstrated that OxPCs were a potent driver of neuronal degeneration in MS pathology [12]. OxPCs accumulated in MS-lesion sites were overlapped with CD45^+^Iba1^+^ cells including microglia and macrophages. In fact, OxPCs clearance from extracellular space mediated by TREM2-expressing microglia/macrophages is necessary to limit progression of neurodegeneration [12].

### Cholesterol

Brain cholesterol accounts for 25% among the whole body, and myelin contains approximately 70% of total cholesterol in the adult brain [60]. In addition to experimental autoimmune encephalomyelitis (EAE) model, cuprizone or LPC treatment is often used to induce demyelination and following remyelination which requires damaged myelin clearance by microglia/macrophages [35]. After phagocytosing myelin debris, newly cholesterol-synthesizing process in microglia is inhibited in response to large amount of intracellular cholesterol. This machinery results in accumulation of desmosterol, the immediate cholesterol precursor, and thus, high levels of desmosterol are commonly observed in EAE model, LPC-injected, and cuprizone-treated mice [1]. Since desmosterol has an agonistic activity at liver X receptor (LXR), LXR-dependent cholesterol efflux genes *Abca1* and *Apoe* are consequently upregulated, which supports remyelination by oligodendrocytes [1].

Unlike young mice (3 months old), aged mice (1 year old) often fail to repair demyelinating lesions after LPC injection [6]. Such lower ability to recover from demyelination is also observed in *Trem2*-deficient mice after cuprizone treatment [43]. Of note, microglia in these mice exhibit an excessive accumulation of LD due to dysregulated expression of genes related to cholesterol metabolism, such as *Abca1*, *Abcg1*, *Acat*, and *Apoe* [6, 17, 39, 43]. Together, these evidences indicate that TREM2 plays a central role in regulating expressions of lipid metabolic genes in microglia.

## Microglial responses against extracellular lipids

Microglia express a broad range of receptors responsive to lipid mediators, such as prostaglandins, endocannabinoids, lysophospholipids, sphingosine-1-phosphate, and others. These bioactive mediators are well characterized in previous studies [2, 13, 51, 58]. One of the well-recognized lipids acting on macrophages/microglia is PS, which is known as “eat-me signal.” Although PS is localized exclusively in the inner leaflet of cell membranes, it can be exposed to cell surface when cells undergo apoptosis. Macrophages/microglia recognize the exposed PS by several PS receptors, such as TIM4, GPR56, and TREM2, followed be engulfing them [31, 36]. Among them, TREM2 can recognize a variety of extracellular phospholipids as well as lipidated-ApoE [55]. The TREM2-ApoE interaction facilitates microglial amyloid-β uptake [14, 28, 59], which may explain why the mutations in *Trem2* and *Apoe* are risk factors of neurological diseases. A recent study demonstrated that β-glucosylceramide, which accumulated in neurons in Gaucher disease, directly activates microglia via its receptor macrophage-inducible C-type lectin (Mincle, *Clec4e*). Moreover, microglia produce tumor necrosis factor in response to *β*-glucosylceramide, which induces neuronal PS exposure resulting in neuronal loss [52]. Thus, microglia sense extracellular lipids, alter intracellular lipid-related gene expressions and lipid metabolism, and perform diverse cellular functions in response to their environment.

## Conclusion and outlook

Until now, our understanding of microglial biology has rapidly progressed with the help of transcriptomic and proteomic techniques. On the other hand, the challenge to analyze microglial lipidome has only just begun, and the same applies to other cell types in the brain. Even with the limited evidence at present, it is reasonable to assume that lipids play essential roles in the diverse functions of microglia. However, there are still a lot of points to be addressed: spatiotemporal lipidome information in microglia at each life stage, their formation and maintenance mechanisms, their interactions with other CNS cell types, and their involvement of pathogenesis. Moreover, recent studies have characterized the properties of CNS border-associated macrophages (CAMs), which are transcriptomically and anatomically distinct from microglia, highlighting the necessity to analyze these cell types separately. Although new analytical technologies with improved sensitivity will be needed, lipidomic approach has great potential to deepen our understanding of the nature of microglia and CAMs during development and diseases.

## Data Availability

Not applicable.

## Abbreviations

ApoE: Apolipoprotein E
BBB: Blood-brain barrier
CAM: CNS border-associated macrophage
CE: Cholesteryl ester
CNS: Central nervous system
EAE: Experimental autoimmune encephalomyelitis
GPL: Glycerophospholipid
HEXB: Hexosaminidase subunit beta
LD: Lipid droplet
LDAM: Lipid-droplet-accumulating microglia
LPC: Lysophosphatidylcholine
LXR: Liver X receptor
Mfsd2a: Major facilitator superfamily domain containing 2a
MS: Multiple sclerosis
OxPC: Oxidized phosphatidylcholine
PLIN2: Perilipin 2
PS: Phosphatidylserine
PUFA: Polyunsaturated fatty acid
SM: Sphingomyelin
SREBP1: Sterol regulatory element-binding protein 1
TREM2: Trigger receptor expressed on myeloid cells 2
